# twas_sim, a Python-based tool for simulation and power analysis of transcriptome-wide association analysis

**DOI:** 10.1093/bioinformatics/btad288

**Published:** 2023-04-26

**Authors:** Xinran Wang, Zeyun Lu, Arjun Bhattacharya, Bogdan Pasaniuc, Nicholas Mancuso

**Affiliations:** Department of Population and Public Health Sciences, Keck School of Medicine, University of Southern California, Los Angeles, CA 90033, United States; Department of Population and Public Health Sciences, Keck School of Medicine, University of Southern California, Los Angeles, CA 90033, United States; Department of Pathology and Laboratory Medicine, David Geffen School of Medicine, University of California, Los Angeles, Los Angeles, CA 90095, United States; Institute of Quantitative and Computational Biosciences, David Geffen School of Medicine, University of California, Los Angeles, Los Angeles, CA 90095, United States; Department of Pathology and Laboratory Medicine, David Geffen School of Medicine, University of California, Los Angeles, Los Angeles, CA 90095, United States; Department of Human Genetics, David Geffen School of Medicine, University of California, Los Angeles, Los Angeles, CA 90095, United States; Department of Computational Medicine, David Geffen School of Medicine, University of California, Los Angeles, Los Angeles, CA 90095, United States; Department of Population and Public Health Sciences, Keck School of Medicine, University of Southern California, Los Angeles, CA 90033, United States; Department of Quantitative and Computational Biology, University of Southern California, Los Angeles, CA 90097, United States

## Abstract

**Summary:**

Genome-wide association studies (GWASs) have identified numerous genetic variants associated with complex disease risk; however, most of these associations are non-coding, complicating identifying their proximal target gene. Transcriptome-wide association studies (TWASs) have been proposed to mitigate this gap by integrating expression quantitative trait loci (eQTL) data with GWAS data. Numerous methodological advancements have been made for TWAS, yet each approach requires *ad hoc* simulations to demonstrate feasibility. Here, we present twas_sim, a computationally scalable and easily extendable tool for simplified performance evaluation and power analysis for TWAS methods.

**Availability and implementation:**

Software and documentation are available at https://github.com/mancusolab/twas_sim.

## 1 Introduction

Genome-wide association studies (GWASs) have identified numerous genetic variants associated with complex traits and diseases (Visscher et al. 2017). However, most associated variants fall within non-coding regions, which makes identifying the target gene challenging (Hindorff et al. 2009; Edwards et al. 2013). Furthermore, functional evidence suggests that most GWAS hits are involved in regulatory processes (Maurano et al. 2012; Vierstra et al. 2020), which implies that causal variants regulate the expression of nearby genes. Transcriptome-wide association studies (TWASs) have been proposed to address this limitation by integrating expression quantitative trait loci (eQTL) data with GWAS data to identify functionally informed gene-level associations (Gamazon et al. 2015; Gusev et al. 2016). A growing ecosystem of methods have been developed around TWAS, each relying on different statistical assumptions (Mancuso et al. 2019; Nagpal et al. 2019; Bhattacharya et al. 2021; Liu et al. 2021; Tang et al. 2021; Lu et al. 2022; Parrish et al. 2022). Prior methodological work evaluated performance through a combination of *ad hoc* simulations and real data analysis. However, validating and assessing model performance requires researchers to implement custom simulations, which duplicates effort and can result in subtle differences in how baselines are defined.

To address this, we developed twas_sim, a computationally scalable and easily extendable tool for downstream TWAS method evaluation and comparison (e.g., statistical power, false positive rate, etc.). It leverages real genetic data to capture typical linkage disequilibrium (LD) patterns and can simulate gene expression levels and complex traits under a variety of feasible genetic architectures. Importantly, it is capable of dynamically loading custom code (e.g., Python, R, and Julia) to evaluate independently developed TWAS methods. It is freely available at https://github.com/mancusolab/twas_sim.

## 2 Implementation

twas_sim is a python-based tool that uses real genotype data to generate TWAS test statistics by simulating complex traits as a function of latent expression levels, fitting eQTL weights in independent reference data, and performing genome- and predicted transcriptome-association testing on the simulated complex trait (see Supplementary Fig. S1). twas_sim accepts optional arguments to vary eQTL/GWAS sample sizes, genetic architectures (e.g., $h_{g}^{2}$, $h_{ge}^{2}$, and sparsity of eQTL effects), horizontal pleiotropy through linkage, and reference genotype datasets for each step in the pipeline (e.g., GWAS, eQTL reference, and TWAS testing). For details on parameters and options, see Supplementary Tables S1 and S2 and Supplementary Note.

twas_sim supports simulating GWAS summary data through two possible modes. Standard mode simulates genotypes for GWAS individuals using multivariate normal approximations parameterized by LD at the genomic region, simulates phenotypes under a fixed eQTL and trait architecture, and finally performs marginal regression at each approximate SNP to obtain GWAS summary statistics. When GWAS sample size, $N_{\mathrm{GWAS}}$, is large, this process requires large amounts of memory (i.e., $O(N_{\mathrm{GWAS}}\cdot P)$, where P is the number of genetic variants. As a workaround, twas_sim supports fast mode, which simulates GWAS summary statistics directly using the multivariate normal distribution parameterized by LD (Pasaniuc and Price 2017). By making distributional assumptions of the underlying summary statistics, this setting bypasses the need for individual-level genotype data and requires memory only proportional to $O(P^{2})$, which can vastly reduce the memory footprint and vastly speed up simulation times (see Supplementary Note). Importantly, to model LD misspecification, twas_sim supports the option to use different LD reference panels across GWAS and eQTL simulations in addition to TWAS testing. To predict gene expression levels into GWAS data, twas_sim supports internally fitting least absolute shrinkage and selection operator (LASSO), elastic net and genomic best linear unbiased prediction (GBLUP) linear prediction models from simulated reference gene expression data; in addition, it also allows users to use true eQTL effect sizes for TWAS calculation instead of regularization method (Searle et al. 1992; Tibshirani 1996; Zou and Hastie 2005). The dynamic import feature enables twas_sim to include external prediction tools easily. It requires only that users define a simple Python interface with a function named “fit” (see Supplementary Note and Supplementary Algorithms S1 and S2). To illustrate the simplicity of our dynamic import approach, we have provided two example scripts in the repository to perform Ordinary Least Square (OLS) regression using sklearn and the Sum of Single Effects (SuSiE) sparse regression from susieR (Wang et al. 2020).

## 3 Application

**Figure 1.**
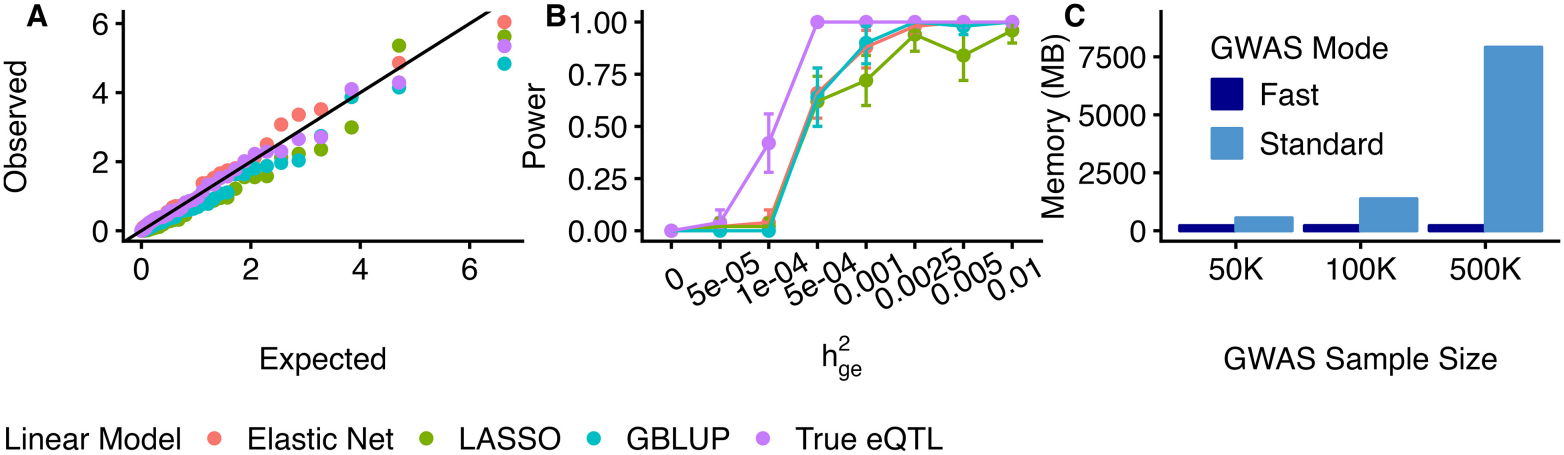
TWAS simulation results. (A) QQ plot for TWAS *χ*^2^ under the null hypothesis. Each point reflects the *χ*^2^ statistic under null simulations based on different predictive models. (B) TWAS power analysis. Each point reflects the proportion of simulations where the null was rejected at *P* < 2.27*E*–06. *X*-axis reflects the proportion of trait variability explained by gene expression (C) Memory usage by simulation mode. Height of bars reflects the average memory usage for fast/standard simulation modes. All error bars reflect 95% confidence interval.

To illustrate the utility of twas_sim, we performed simulations using genetic data from 1000Genomes (1000 Genomes Project Consortium 2015) across a variety of gene expression and complex trait architectures, and genotype reference panels (see Supplementary Note). First, we investigated unbiasedness under null simulations (i.e., *α* = 0) under three metrics: Kolmogorov–Smirnov test on TWAS *Z*-scores, family-wise error rate (FWER) on TWAS *P*-value, and inflation (see Supplementary Note). We found TWAS test statistics computed using Elastic Net are largely consistent with the null (*P* = 0.26) and observed similar patterns for other linear models (see Fig. 1A). Focusing on Elastic Net prediction models, we observed similar results under various eQTL architectures, eQTL/GWAS sample sizes, and simulation modes (see Supplementary Table S3). Next, we evaluated FWER with found calibrated results across prediction models, eQTL architecture, eQTL/GWAS sample sizes, and simulation modes (see Supplementary Fig. S2). Similarly, we found no inflation across all settings (see Supplementary Fig. S3). Together, these results suggest that TWAS test statistics are robust to model assumptions.

Next, we evaluated the power of each prediction model when a causal relationship between eQTL and complex trait exists (i.e., *α* ≠ 0). We observed Elastic Net (power = 0.66) outperformed GBLUP (power = 0.64), LASSO (power = 0.62), and SuSiE (power = 0.44; see Supplementary Figs S4 and S5). We assessed power under various simulation settings and observed power increased with increasing $h_{ge}^{2}$, GWAS and eQTL sample sizes, eQTL and sparsity of eQTL architectures (see Fig. 1B; Supplementary Figs S4 and S5).

To assess the degree to which LD misspecification affects TWAS test statistics, we performed simulations splitting 1000G EUR individuals into two subsets (*N* = 244, 245). The first subset was used to simulate GWAS test statistics, whereas the second was used for eQTL simulation and downstream TWAS testing. Under the null, we found TWAS test statistics computed using the same reference panel (*P *=* 0*.26) and the misspecified reference panel (*P *=* 0*.57) were largely consistent (see Supplementary Table S3), with similar estimates inflation (*P* = 0.049) and moderately reduced FWER (*P* = 0.005). In simulations under a causal model, we observed LD misspecification reduced power significantly compared with the correctly specified model (*P *=* *2.2*E*–16; see Supplementary Fig. S6A–L).

To highlight the scalability of twas_sim to extremely large GWAS sample sizes, we evaluated its performance under standard and fast simulation modes. We found fast mode required 6× and 36× less memory and 8× and 41× less CPU time compared with standard mode, for GWAS sample sizes of 100K and 500K, respectively (see Fig. 1C and Supplementary Fig. S7).

Lastly, to assess how horizontal pleiotropy through linkage (i.e., genes whose eQTLs are in LD with eQTLs for a causal gene) inflates TWAS test statistics, we simulated GWAS effect sizes independently from eQTLs and performed TWAS testing. Overall, we found that while TWAS test statistics at tagging genes were not as large as those computed using the causal gene (see Supplementary Fig. S8A), we observed significantly inflated test statistics resulting in an elevated FWER (*P *= 0.02), which is consistent with previous works (Mancuso et al. 2019; Wainberg et al. 2019; Lu et al. 2022) emphasizing the need for joint testing of multiple nearby genes or statistical fine-mapping (see Supplementary Note and Supplementary Fig. S8B).

## 4 Conclusion

Here, we present twas_sim, a flexible and scalable computational simulation tool of TWAS test statistics. It simulates expression levels and complex traits under a variety of feasible genetic architectures. Simulation results are easily interpretable for downstream model evaluation. The simulator currently supports fitting LASSO, Elastic Net, and GBLUP prediction models to predict gene expression into GWAS. It is easily extendable with dynamic import function to include additional linear models to accommodate TWAS methods.

## Supplementary Material

btad288_Supplementary_Data
