# Supplementary material for: twas_sim, a Python-based tool for simulation and power analysis of transcriptome-wide association analysis
Source: Bioinformatics. 2023 Apr 26;39(5):btad288. doi: 10.1093/bioinformatics/btad288 (PMC10172036; doi:10.1093/bioinformatics/btad288)
Supplement: btad288_Supplementary_Data [file btad288_supplementary_data.docx]

­Supplementary Materials: twas_sim, a Python-based tool for simulation and power analysis of transcriptome-wide association analysis

Xinran Wang^1,*^, Zeyun Lu^1^, Arjun Bhattacharya^2,3^, Bogdan Pasaniuc^2,4,5^, and Nicholas Mancuso^1,6*^

1. Department of Population and Public Health Sciences, Keck School of Medicine, University of Southern California, Los Angeles, CA
2. Department of Pathology and Laboratory Medicine, David Geffen School of Medicine, University of California, Los Angeles, Los Angeles, CA, USA
3. Institute of Quantitative and Computational Biosciences, David Geffen School of Medicine, University of California, Los Angeles, Los Angeles, CA, USA
4. Department of Human Genetics, David Geffen School of Medicine, University of California, Los Angeles, Los Angeles, CA, USA
5. Department of Computational Medicine, David Geffen School of Medicine, University of California, Los Angeles, Los Angeles, CA, USA
6. Department of Quantitative and Computational Biology, University of Southern California, Los Angeles, CA

- To whom correspondence should be addressed.

# Supplementary Note

**Model for gene expression and complex phenotype.** The first step of simulating TWAS association statistics is to compute genotype matrix and β coefficient for eQTL panel. We use reference linkage-disequilibrium (LD) data from 1000Genomes (Sudmant *et al.*, 2015; 1000 Genomes Project Consortium *et al.*, 2015) to simulate genotype samples and user-defined architecture to simulate eQTL reference panel. Then, we construct models for gene expression and complex trait. We use a linear function of genotype matrix ${\mathbf{X}\mathbb{\in R}}^{n\boldsymbol{\times}p}$ for $n$ individuals and $p$ SNPs, to model gene expression level ${\mathbf{g}\mathbb{\in R}}^{n}$ as,

$$\mathbf{g}=\mathbf{X}\boldsymbol{\beta}_{\mathrm{eQTL}}+\boldsymbol{\varepsilon}_{ge}$$

where ${\boldsymbol{\beta}_{\mathrm{eQTL}}\mathbb{\in R}}^{p}$ is the vector for eQTL effect size, $\boldsymbol{\varepsilon}_{ge}\boldsymbol{\sim}\mathbf{N}\mathbf{(}\mathbf{0}\mathbf{,}\sigma_{ge}^{2}\mathbf{I}_{n})$ is random environmental noise such that $\sigma_{ge}^{2}:=1-h_{g}^{2}$, and $h_{g}^{2}$ is the SNP-heritability of gene expression (Shi *et al.*, 2016). We use a linear function of expression levels **g** to model $\mathbf{y}\mathbb{\in R}^{n}$, the normally distributed quantitative trait for $n$ individuals as

$$\mathbf{y}=\mathbf{g}\alpha+\boldsymbol{\varepsilon}\boldsymbol{=}\left( \mathbf{X}\boldsymbol{\beta}_{\mathrm{eQTL}}+\boldsymbol{\varepsilon}_{ge} \right)\alpha\boldsymbol{+}\boldsymbol{\varepsilon}\boldsymbol{=}\mathbf{X}\boldsymbol{\beta}_{\mathrm{eQTL}}\alpha+\left( \boldsymbol{\varepsilon}_{ge}\alpha\boldsymbol{+}\boldsymbol{\varepsilon} \right)$$

$$\boldsymbol{=}\mathbf{X}\boldsymbol{\beta}_{\mathrm{eQTL}}\alpha+\boldsymbol{\varepsilon}_{y}$$

$$\mathbf{=X}\boldsymbol{\beta}_{\mathrm{GWAS}}+\boldsymbol{\varepsilon}_{y}\boldsymbol{,}$$

where $\alpha$ is the causal effect of gene expression, $\boldsymbol{\varepsilon}_{y}\boldsymbol{\sim}\mathbf{N}\mathbf{(}\mathbf{0}\mathbf{,}\sigma_{y}^{2}\mathbf{I}_{n})$ is random environmental noise such that $\sigma_{y}^{2}:=1-h_{ge}^{2}$ where $h_{ge}^{2}$ is proportion of variance explained in complex trait $\mathbf{y}$ by the cis-genetic component of gene expression levels **g**.

**Model for genome-wide association study summary statistics and fast GWAS simulation.** We simulate GWAS summary statistics using two different modes. The *standard* mode simulates genotypes for GWAS individuals using multi-variate normal approximations parameterized by estimated linkage disequilibrium (LD) at the genomic region, simulates phenotypes under a fixed eQTL and trait architecture using the above equations, and finally performs marginal regression at each approximate SNP to obtain GWAS summary statistics. Here, we describe how to perform GWAS simulations without generating simulated individual-level data (i.e. *fast* mode). Given that $\boldsymbol{\beta}_{\mathrm{GWAS}}=\boldsymbol{\beta}_{\mathrm{eQTL}}\alpha$ (see above), using standard linear regression we can compute ${\hat{\boldsymbol{\beta}}}_{\mathrm{GWAS}}$ for $j^{th}$ SNP as

$$\hat{\beta}_{GWAS, j}={(\mathbf{x}_{j}^{T}\mathbf{x}_{j})}^{-1}\mathbf{x}_{j}^{T}\mathbf{y}\approx\frac{1}{n}\mathbf{x}_{j}^{T}\mathbf{y,}$$

where $\mathbf{x}_{j}$ is centered and standardized genotype vector at the $j^{th}$ SNP. Thus, we can compute the entire vector of marginal estimates as

$${\hat{\boldsymbol{\beta}}}_{\mathrm{GWAS}}\approx\frac{1}{n}\mathbf{X}^{T}\mathbf{y,}$$

where ${\mathbf{X}\mathbb{\in R}}^{n\times p}$ is centered and standardized genotype matrix at all $p$ SNPs. Alternatively, we can model GWAS summary statistics directly using a multivariate normal distribution parameterized by estimated LD and causal effects $\boldsymbol{\beta}_{\mathrm{GWAS}}$, which is a common modelling assumption in numerous statistical genetic frameworks (Pasaniuc and Price, 2017; Yang *et al.*, 2012). Based on the computation above, we can compute the expectation and variance of ${\hat{\boldsymbol{\beta}}}_{\mathrm{GWAS}}$ as:

$$\mathbb{E}\left[ {\hat{\boldsymbol{\beta}}}_{\mathrm{GWAS}} \right]\mathbb{=E}\left[ \frac{1}{n}\mathbf{X}^{T}\mathbf{y} \right]=\frac{1}{n}\mathbf{X}^{T}\mathbb{E}\left[ \mathbf{y} \right]=\frac{1}{n}\mathbf{X}^{T}\mathbf{X}\boldsymbol{\beta}_{\mathrm{GWAS}}=\mathbf{V}\boldsymbol{\beta}_{\mathrm{GWAS}}$$

$$\mathbb{V}\left[ {\hat{\boldsymbol{\beta}}}_{\mathrm{GWAS}} \right]\mathbb{=V}\left[ \frac{1}{n}\mathbf{X}^{T}\mathbf{y} \right]=\frac{1}{n}\mathbf{X}^{T}\mathbb{V}\left[ \mathbf{y} \right]\mathbf{X}\frac{1}{n}=\frac{1}{n^{2}}\mathbf{X}^{T}\left( \mathbf{I}_{n}\sigma_{y}^{2} \right)\mathbf{X}=\frac{\sigma_{y}^{2}}{n}\frac{1}{n}\mathbf{X}^{T}\mathbf{X}=\frac{\sigma_{y}^{2}}{n}\mathbf{V}$$

where $\mathbf{V}$ is the $p\times p$ LD matrix. Together we have,

$${\hat{\boldsymbol{\beta}}}_{\mathrm{GWAS}} \sim N\left( \mathbf{V}\boldsymbol{\beta}_{\mathrm{GWAS}}\mathbf{,V}\frac{\sigma_{y}^{2}}{n} \right).$$

Typically, $n$ is large and the normal distribution is a good approximation. However, we extend our framework to the $t$ distribution to allow for additional variability at low sample sizes. Specifically, we use an inverse gamma distribution to model the variance in the GWAS effect estimate at the $j^{\text{th}}$ SNP as

$$s_{j}^{2} \sim\Gamma^{-1}\left( \frac{v}{2}, \frac{v\tau^{2}}{2} \right),$$

where $v=n-1 \mathrm{and} \tau^{2}={\sigma_{y}^{2}}/n$. Let $\mathbf{D}\mathbf{=}\mathbf{diag}\mathbf{(}s_{1}, \ldots, s_{p})$ be a diagonal matrix of GWAS standard errors, then we sample GWAS summary data following ref (Zhu and Stephens, 2017) as,

${\hat{\boldsymbol{\beta}}}_{\mathrm{GWAS}}\sim N\left( \mathbf{D}\mathbf{V}\mathbf{D}^{\mathbf{-}\mathbf{1}}\boldsymbol{\beta}_{\mathrm{GWAS}}\mathbf{,}\mathbf{D}\mathbf{VD} \right)$.

**Gene expression prediction models.** We use LASSO, Elastic Net, and GBLUP predictive penalized linear models to fit effect sizes ${\hat{\mathbf{w}}}_{\mathrm{Lasso}}$, ${\hat{\mathbf{w}}}_{\mathrm{EN}}$, and ${\hat{\mathbf{w}}}_{\mathrm{GBLUP}}$ (Tibshirani, 1996; Zou and Hastie, 2005; Hoerl and Kennard, 1970; Patterson and Thompson, 1971; Goeman, 2010). We use $\hat{\boldsymbol{w}}$ to represent penalized predicative weights instead of ${\hat{\boldsymbol{\beta}}}_{\boldsymbol{eQTL}}$. We compute LASSO predictive model as:

$${\hat{\mathbf{w}}}_{\mathrm{Lasso}}:=\mathrm{argmin}_{\mathbf{w}} \left\| \mathbf{g}-\mathbf{X}\mathbf{w} \right\|_{2}^{2}+\frac{\lambda_{1}}{2}\left\| \mathbf{w} \right\|_{1}$$

using L1 penalized regression, where $\lambda_{1}$ is the coefficient shrinkage adjustment constant. We compute Elastic Net predictive model as

$${\hat{\mathbf{w}}}_{\mathrm{EN}}:=\mathrm{argmin}_{\mathbf{w}} \left\| \mathbf{g}-\mathbf{X}\mathbf{w} \right\|_{2}^{2}+\frac{\lambda_{2}\lambda_{1}}{2}\left\| \mathbf{w} \right\|_{1}+\frac{\left( 1-\lambda_{2} \right)\lambda_{1}}{2}\left\| \mathbf{w} \right\|_{2}^{2}$$

using L1/L2 penalized regression, where $\lambda_{2}$ is the weight that tradeoff between 0 and 1. We compute

GBLUP predictive model as

$${\hat{\mathbf{w}}}_{\mathrm{GBLUP}}:=\mathrm{argmin}_{\mathbf{w}} \left\| \mathbf{g}-\mathbf{X}\mathbf{w} \right\|_{2}^{2}+\lambda_{1}\left\| \mathbf{w} \right\|_{2}^{2}$$

using penalty computed using REML variance component estimates (Searle *et al.*, 1992) as ${{\lambda_{1}=\sigma}_{ge}^{2}}/{({h_{g}^{2}}/p)}.$

**Model for Transcriptome-Wide Association Study.** According to TWAS (Gusev *et al.*, 2016; Gamazon *et al.*, 2015), we can first estimate the causal effect of gene expression, $\hat{\alpha}$, using ordinary least squares (OLS) regression as,

$$\hat{\alpha}=\frac{{\hat{\mathbf{g}}}^{T}\mathbf{y}}{\left( {\hat{\mathbf{g}}}^{T}\hat{\mathbf{g}} \right)}=\frac{\left( \mathbf{X}\hat{\mathbf{w}} \right)^{T}\mathbf{y}}{(\mathbf{X}{\hat{\mathbf{w}})}^{T}\mathbf{X}\hat{\mathbf{w}}}=\frac{{\hat{\mathbf{w}}}^{T}\mathbf{X}^{T}\mathbf{y}}{n{\hat{\mathbf{w}}}^{T}\mathbf{V}\hat{\mathbf{w}}}$$

where $\hat{\mathbf{g}}$ is the predicted gene expression. We can compute the variance of the estimation of causal effect of gene expression ($\hat{\alpha}$) as,

$$\mathbb{V}\left[ \hat{\alpha} \right]=\mathbb{V}\left[ \frac{{\hat{\mathbf{w}}}^{T}\mathbf{X}^{T}\mathbf{y}}{n{\hat{\mathbf{w}}}^{T}\mathbf{V}\hat{\mathbf{w}}} \right]=\frac{{\hat{\mathbf{w}}}^{T}\mathbf{X}^{T}}{n{\hat{\mathbf{w}}}^{T}\mathbf{V}\hat{\mathbf{w}}}\mathbb{V}\left[ \mathbf{y} \right]\frac{\mathbf{X}\hat{\mathbf{w}}}{n{\hat{\mathbf{w}}}^{T}\mathbf{V}\hat{\mathbf{w}}}=\frac{\sigma_{y}^{2}}{n{\hat{\mathbf{w}}}^{T}\mathbf{V}\hat{\mathbf{w}}}.$$

Lastly, we compute TWAS Z-score as,

$$z_{TWAS}=\frac{\hat{\alpha}}{\sqrt{\mathbb{V}\left[ \hat{\alpha} \right]}}=\frac{{\hat{\mathbf{w}}}^{T}\mathbf{X}^{T}\mathbf{y}}{n{\hat{\mathbf{w}}}^{T}\mathbf{V}\hat{\mathbf{w}}} \cdot\frac{\sqrt{n{\hat{\mathbf{w}}}^{T}\mathbf{V}\hat{\mathbf{w}}}}{\sigma_{y}}=\frac{{\hat{\mathbf{w}}}^{T}\mathbf{z}_{\mathrm{GWAS}}}{\sqrt{{\hat{\mathbf{w}}}^{T}\mathbf{V}\hat{\mathbf{w}}}},$$

where $\mathbf{z}_{\mathrm{GWAS}}:=\frac{1}{\sqrt{n}\sigma_{y}}\mathbf{X}^{T}\mathbf{y}$.

**Simulation data preparation.** Here we describe our simulation pipeline that includes reference genotype data pre-processing and twas_sim. To simulate a region with sufficient complexity, we first sample a genomic region uniformly at random, among approximately independent LD blocks that harbor between 5-20 genes, using RefSeq gene definitions (O’Leary *et al.*, 2016; Berisa and Pickrell, 2016). Next, we subset 1000G reference genotype data (1000 Genomes Project Consortium *et al.*, 2015) from European ancestry individuals to the genomic region from the previous step, while filtering out genetic variants that are not bi-allelic SNPs, have minor allele frequency less than 1%, have Hardy-Weinberg p-value < 1e-5, and variant missingness > 10%. We additionally restrict to HapMap3 variants (International HapMap 3 Consortium *et al.*, 2010). Next, we provide this QC’d reference genotype data to twas_sim to perform simulations under a variety of eQTL and complex trait architectures, sample sizes, and linear prediction models.

**Simulation groups.** We performed two groups of simulations to account for LD misspecification:

- Correct reference panel: use 1000Genomes reference genotypes from all 489 individuals of European ancestry to compute GWAS, eQTL, and TWAS LD information.
- Misspecified reference panel: randomly assign 1000Genomes reference genotypes from 489 individuals of European ancestry into two subgroups, with 244 individuals in the first group and 245 individuals in the second group. Use the reference genotype data from the first subgroup to compute GWAS LD information and the genotype data from the second subgroup to compute eQTL and TWAS LD information.

Additionally, we performed two groups of simulations to generate model $\beta_{GWAS}$ under:

- Causal TWAS Model: dependent GWAS and eQTL signals, and
- Horizontal Pleiotropy through Linkage: independent GWAS and eQTL signals (see **Supplementary Notes)**.

**Parameters**. Here, we define a set of “canonical” parameters that represent a baseline from which we deviated when exploring specific parameter settings,

- GWAS simulation mode: *fast* mode,
- linear model: Elastic Net,
- SNP model: 1% SNPs,
- eQTL sample size: 250,
- GWAS sample size: 200K,
- $h_{ge}^{2}$: 0.0005,
- $h_{g}^{2}$*:* 0.1.

When exploring specific parameter settings, we varied individual parameter as,

- GWAS simulation mode: *fast* or *standard* modes,
- linear model: LASSO, Elastic Net, GBLUP, SuSiE, and True eQTL,
- SNP model: 1 SNP, 1% SNPs, 10% SNPs,
- eQTL sample size: 100, 250, or 500,
- GWAS sample size: 50k, 100k, 200k, 500k,
- $h_{ge}^{2}$: 0.0 (null), 0.00005, 0.0001, 0.0005, 0.001, 0.0025, 0.005, or 0.01,
- $h_{g}^{2}$*:* 0.1.

We perform 50 simulations for each fixed set of simulation parameters.

**Performance metrics and definitions.** We evaluated the performance of twas_sim in terms of unbiasedness, power, degree of LD misspecification, memory usage, and CPU time usage. We investigated unbiasedness from 3 metrics (Kolmogorov-Smirnov test, family-wise error rate, and inflation) under the null hypothesis, which states that there is no association between complex trait and predicted gene expression (i.e., $\alpha=0$). We first used a two-sided, one-sample Kolmogorov-Smirnov (KS) test to test if there was a statistically significant difference between TWAS Z-score distribution and normal distribution for each fixed set of simulation parameters (50 simulations per set) in each simulation group. Second, we calculated family-wise error rate (FWER) to assess the proportion of TWAS null hypothesis being rejected under the null. For each fixed set of simulation parameters, we performed bootstraps with 1,000 repeats to track the proportion of genes that are TWAS significant with nominal P-value < 0.05. Then, we calculated the FWER by computing the mean and 95% confidence interval of the bootstrapped results. Third, we calculated inflation to measure how much twas_sim results deviate from the expected distribution (χ^2^ distribution with 1 degree of freedom). We estimated the inflation and its 95% confidence interval using bootstraps with 1,000 repeats. Specifically, we defined inflation as the median(twas.χ^2^)/ 0.455, where 0.455 is the median of 1-df χ^2^ distribution; in other words, the inflation is expected to be 1 if the twas_sim results is perfectly unbiased.

We defined power as probability of detecting the association between complex trait and predicted gene expression when the association truly exists. In other words, it is the proportion of TWAS alternative hypothesis being accepted under the alternative hypothesis. For each fixed set of simulation parameters, we estimated the power and its 95% confidence interval by performing 1,000 nonparametric bootstraps with the threshold TWAS p-value < 0.05/22,000, where 22,000 represent an approximation of the total number of protein-coding genes within human genome.

We defined LD misspecification as the inconsistency of LD patterns for individuals of the same ancestry within the same reference panel and horizontal pleiotropy through linkage as the situation when nearby tagging genes are also tested in TWAS.

We extracted MaxRSS, the maximum memory usage of each simulation process, from Slurm Workload Manage output files using *sccat* command. In addition, we obtained CPU time usage by *process_time* function from python *time* library.

**Dynamic import of external modules.** To enhance the flexibility of twas_sim and enable the use of externally defined prediction models, we have implemented a dynamic import function that permits users to call external models in a language-agnostic way, provided it is wrapped inside a Python function named `fit`. As a demonstration of this feature, we implemented a module that fits the genetic component of gene expression using the Sum of Single Effects (SuSiE) model (Wang *et al.*, 2020) as implemented in susieR (see **Supplementary Algorithms 1, 2**).

# Supplementary Figures


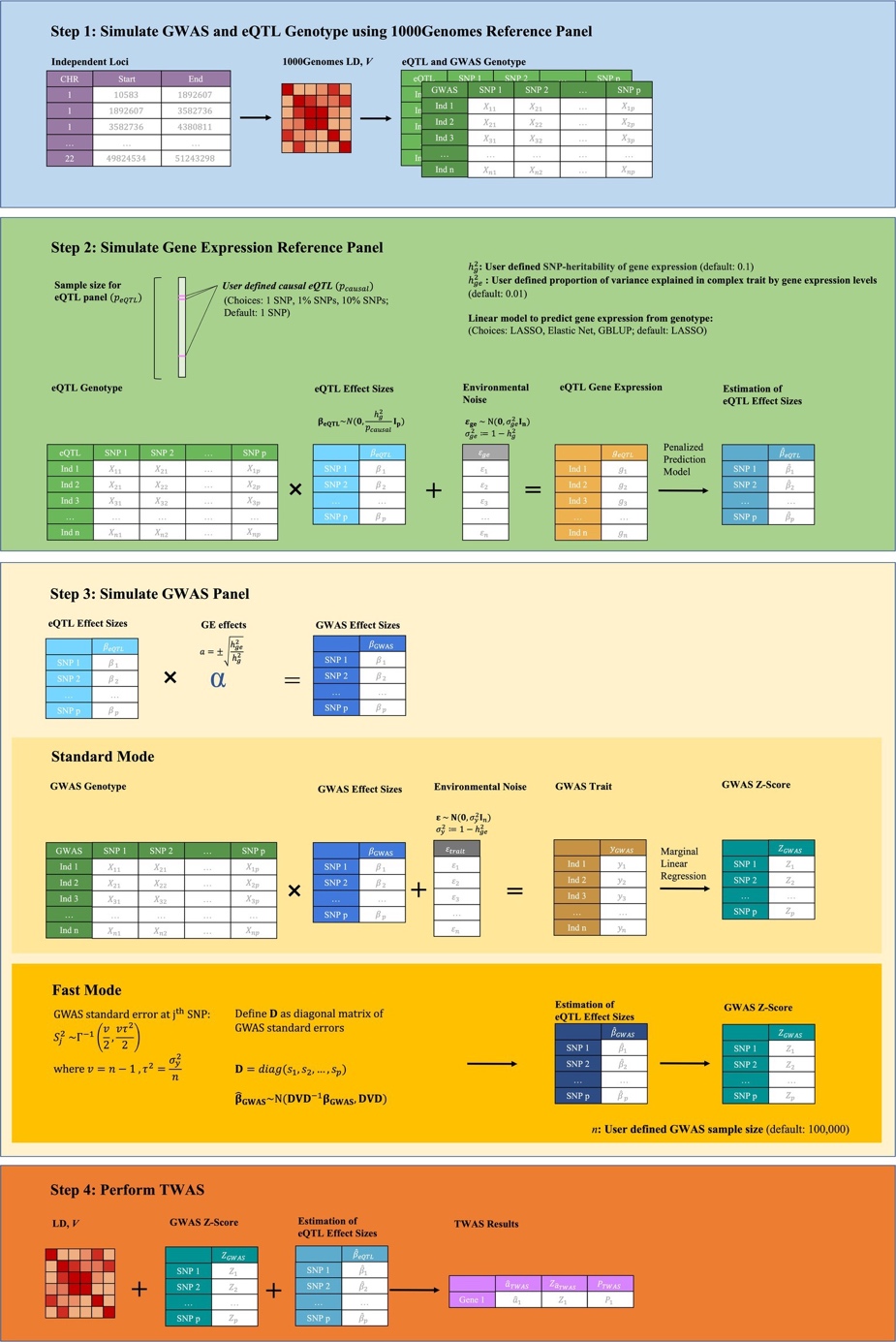


**Supplementary Figure 1. twas_sim workflow.** **Step 1:** We approximated genotypes under 1,000 Genomes reference LD structure using an MVN model. **Step 2:** First, we simulated eQTL effect sizes ($\boldsymbol{\beta}_{\mathrm{eQTL}}$) based on user-defined genetic architectures and environmental noise. Then, we simulated gene expression in eQTL dataset ($\mathbf{g}_{\mathrm{eQTL}}$). Third, we estimated eQTL effect sizes (${\hat{\boldsymbol{\beta}}}_{\mathrm{eQTL}}$) using penalized prediction models (LASSO, Elastic Net, GBLUP). **Step 3:** First, we obtained gene expression effect sizes (α). Second, we calculated GWAS effect sizes ($\boldsymbol{\beta}_{\mathrm{GWAS}}$). Third, we performed GWAS using either *standard* mode or memory efficient *fast* mode. In the *standard* mode, we first generated genotype matrix ($\mathbf{X}_{\mathrm{GWAS}}$) and phenotype ($\mathbf{y}_{\mathrm{GWAS}}$). Then, we regressed phenotype ($\mathbf{y}_{\mathrm{GWAS}}$) using marginal linear regression for each simulated variant to get GWAS Z-score. In the *fast* mode, we simulated GWAS effect size (${\hat{\boldsymbol{\beta}}}_{\mathrm{GWAS}}$) from MVN approximation and generated GWAS Z-score. **Step 4:** We computed TWAS test statistics using LD, GWAS Z-score, and estimated eQTL effect sizes (${\hat{\boldsymbol{\beta}}}_{\mathrm{eQTL}}$).


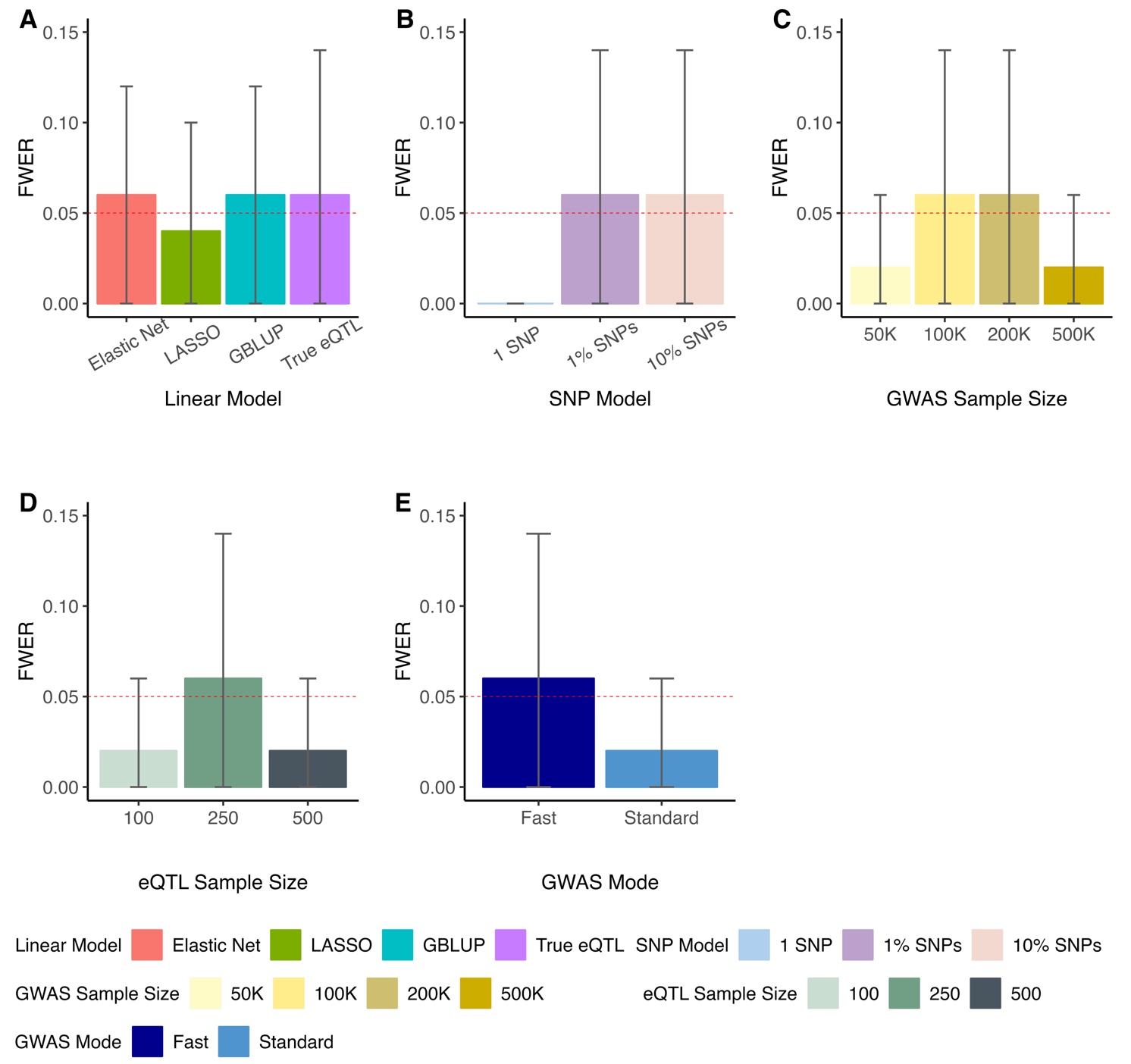


**Supplementary Figure 2. The TWAS statistics simulated by twas_sim is well controlled at P=0.05 under the null in family-wise error rate (FWER).** The bar plot for TWAS FWER (under the null scenario where gene expression effect size α=0) for canonical parameters with various **A.** linear models **B.** SNP models **C.** increasing GWAS sample sizes **D.** eQTL sample sizes **E.** GWAS mode. See **Supplementary Note** for FWER calculation. 50 simulations were performed for each fixed set of simulation parameters. The FWER and its error bars (95% confidence interval) are estimated using bootstraps with 1,000 repeats.

**
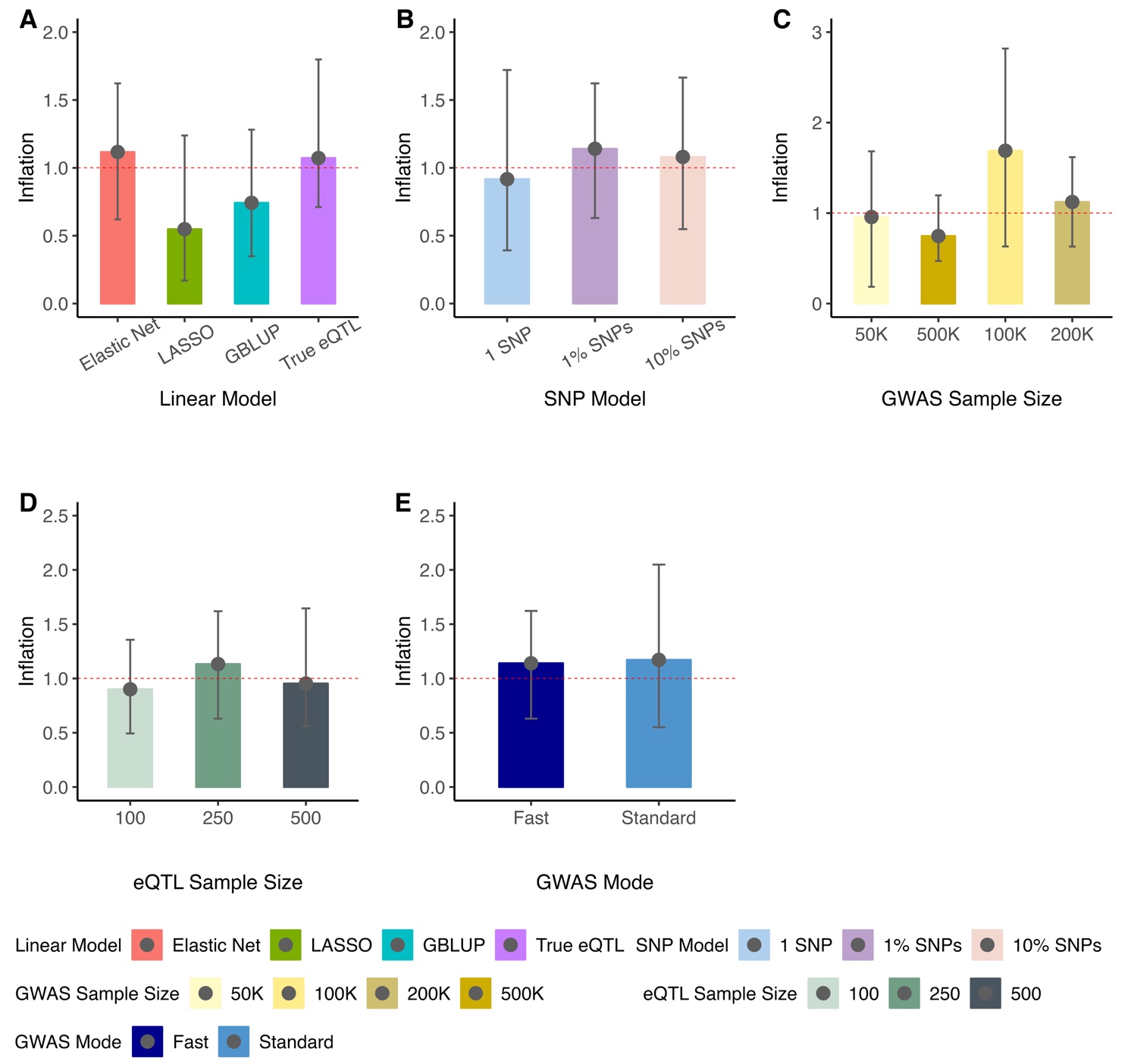
**

**Supplementary Figure 3. twas_sim simulates non-inflated TWAS statistics under the null across eQTL/GWAS sample sizes and genetic architecture**. The bar plot for TWAS inflation (under the null scenario where gene expression effect size α=0) for canonical parameters with various **A.** linear models **B.** SNP models **C.** increasing GWAS sample sizes **D.** eQTL sample sizes **E.** GWAS mode. See **Supplementary Note** for inflation calculation. 50 simulations were performed for each fixed set of simulation parameters. 50 simulations were performed for each fixed set of simulation parameters. The inflation and its error bars (95% confidence interval) are estimated using bootstraps with 1,000 repeats.

**
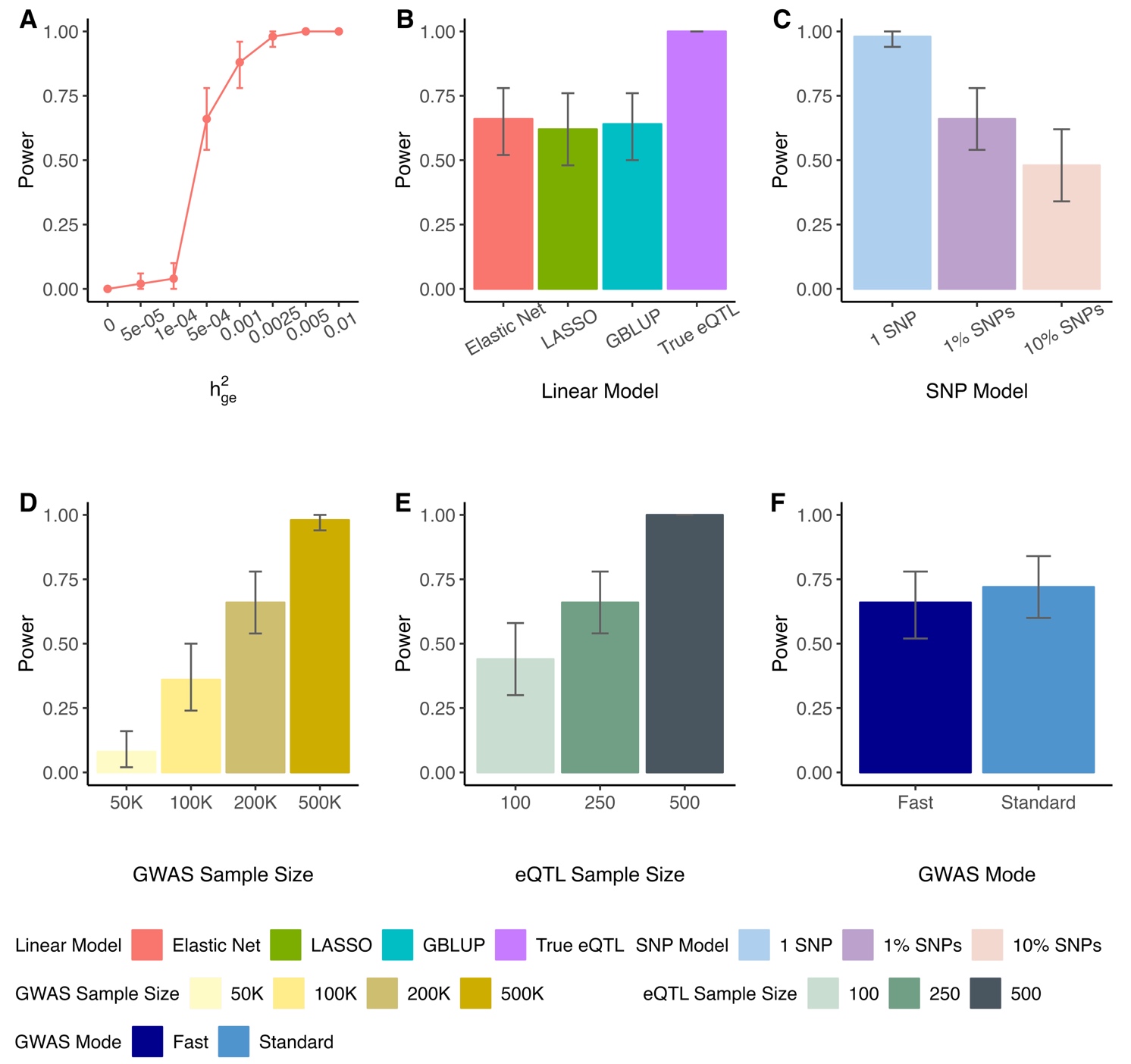
**

**Supplementary Figure 4. The TWAS statistics simulated by twas_sim retain high power under the alternative across eQTL/GWAS sample sizes and genetic.** The plot for TWAS power (under the alternative scenario where gene expression effect size α≠0) for canonical parameters with various **A.** $h_{\mathrm{ge}}^{2}$ **B.** linear models C. SNP models **D.** increasing GWAS sample sizes **E.** eQTL sample sizes **F.** GWAS mode. See **Supplementary Note** for power calculation. 50 simulations were performed for each fixed set of simulation parameters. The power and its error bars (95% confidence interval) are estimated using bootstraps with 1,000 repeats.

**
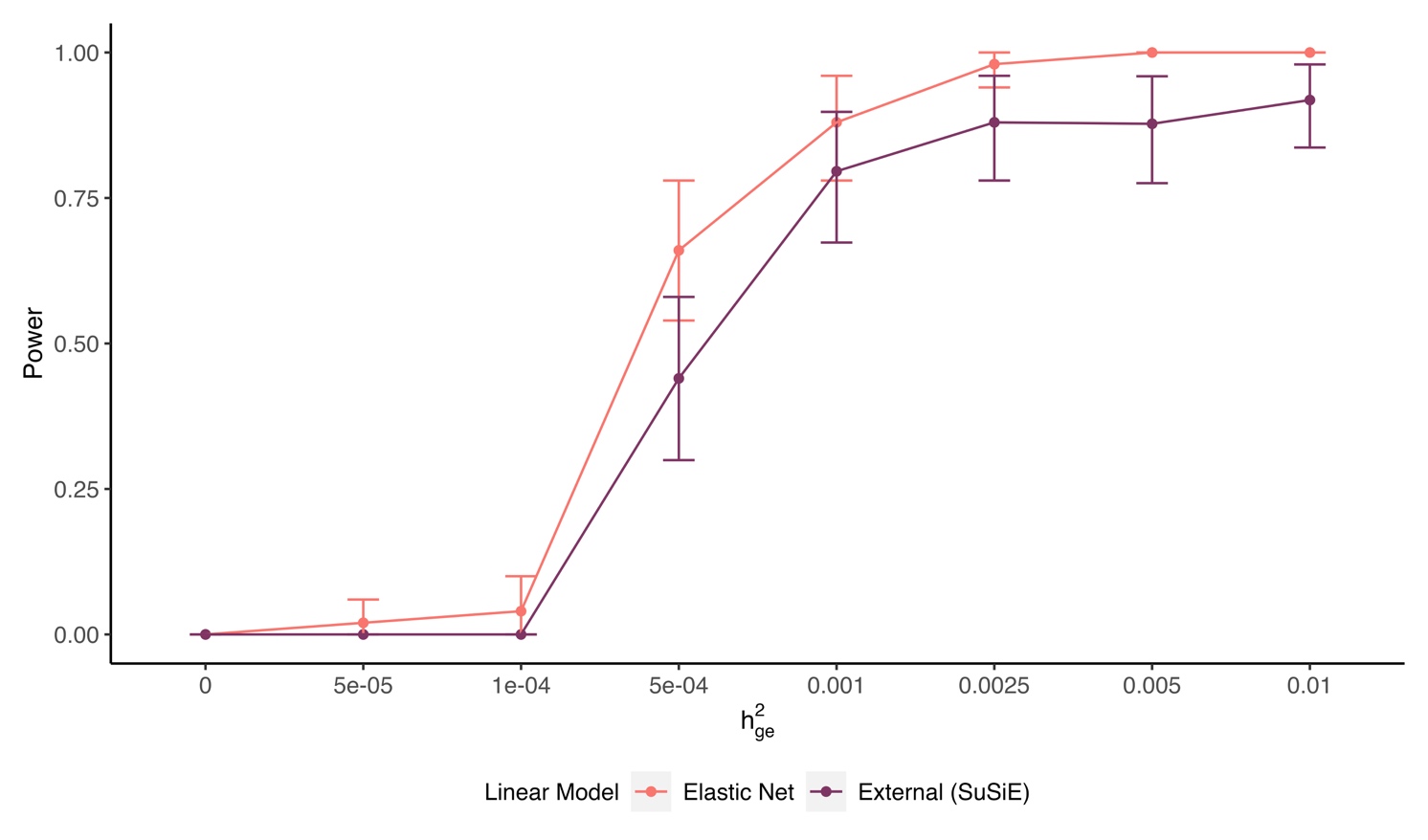
**

**Supplementary Figure 5. SuSiE and Elastic Net has comparable power.** The line plot for TWAS power (under the alternative scenario where gene expression effect size α≠0) for Elastic Net and SuSiE with canonical parameters and increasing $h_{\mathrm{ge}}^{2}$. 50 simulations were performed for each fixed set of simulation parameters. The power and its error bars (95% confidence interval) are estimated using bootstraps with 1,000 repeats.


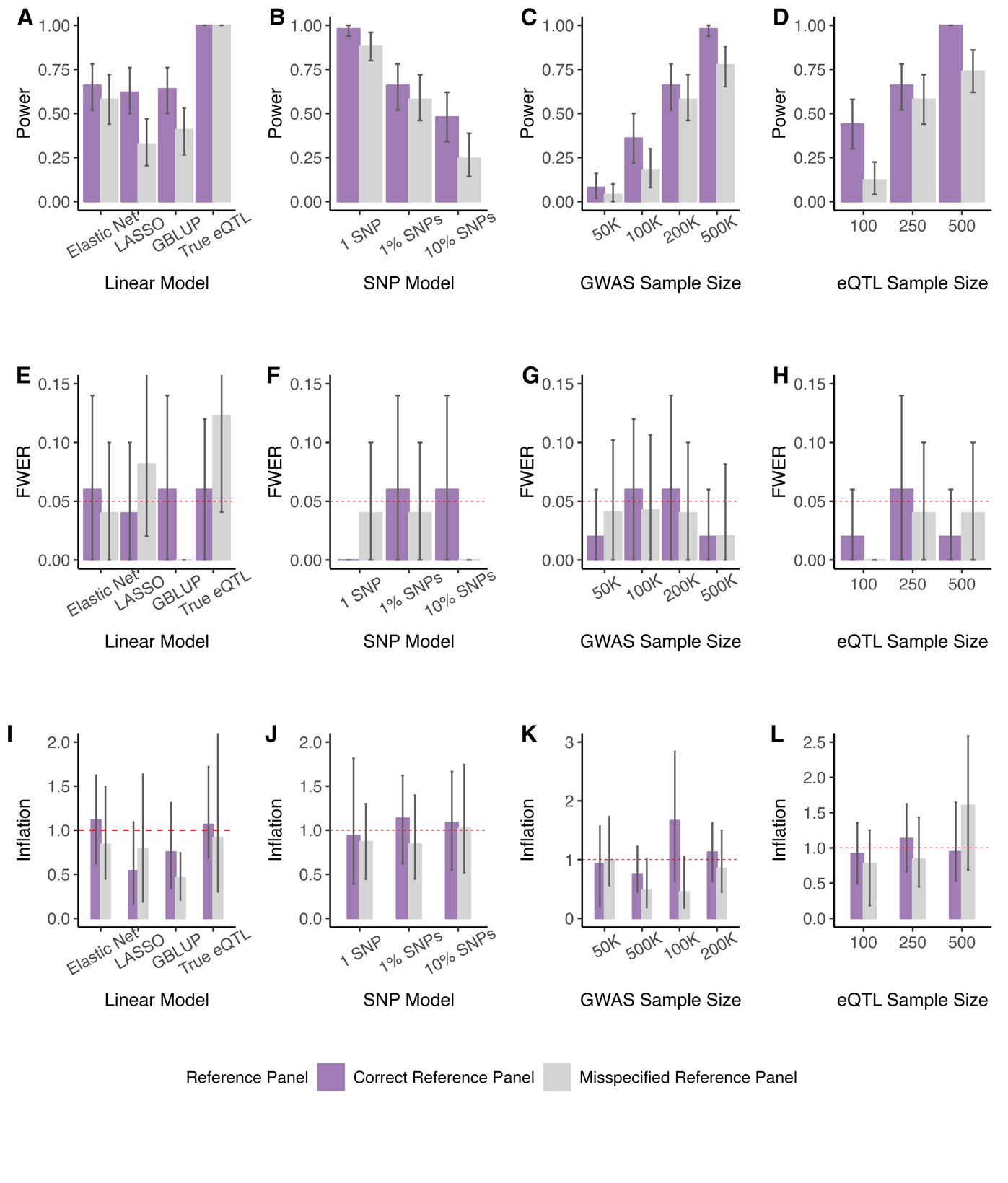


**Supplementary Figure 6. Impact of LD misspecification on statistical power.** We quantified the impact of LD misspecification. Briefly, we performed simulations by splitting all Europeans in 1000G reference panel into two equally divided groups, simulated GWAS and eQTL data using the first LD group, and computed TWAS test statistics using the second LD group. We compared results under this scenario with results obtained using the correctly specified panel (eQTL, GWAS, and TWAS data generated from all Europeans in 1000G reference panel) to assess statistical power (A-D), FWER (E-H), and inflation under the null (I-L). The power, FWER, and inflation and their error bars (95% confidence interval) are estimated using bootstraps with 1,000 repeats.

**
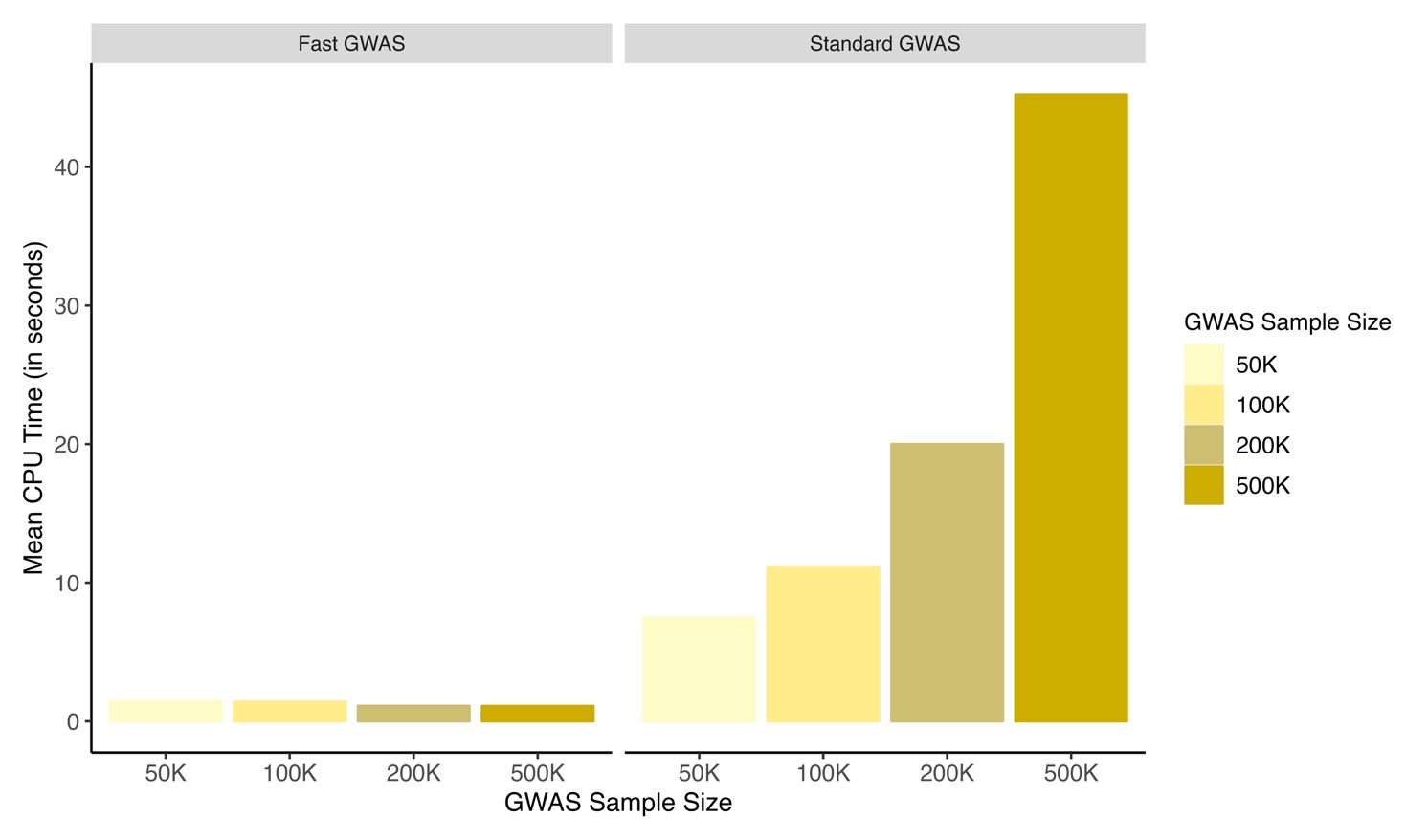
**

**Supplementary Figure 7. *fast* mode outperforms *standard* mode in mean CPU time regardless of GWAS sample sizes.** The bar plot for mean CPU time (in seconds) for canonical parameters with two GWAS modes while increasing GWAS sample sizes. 50 simulations were performed for each fixed set of simulation parameters.

**
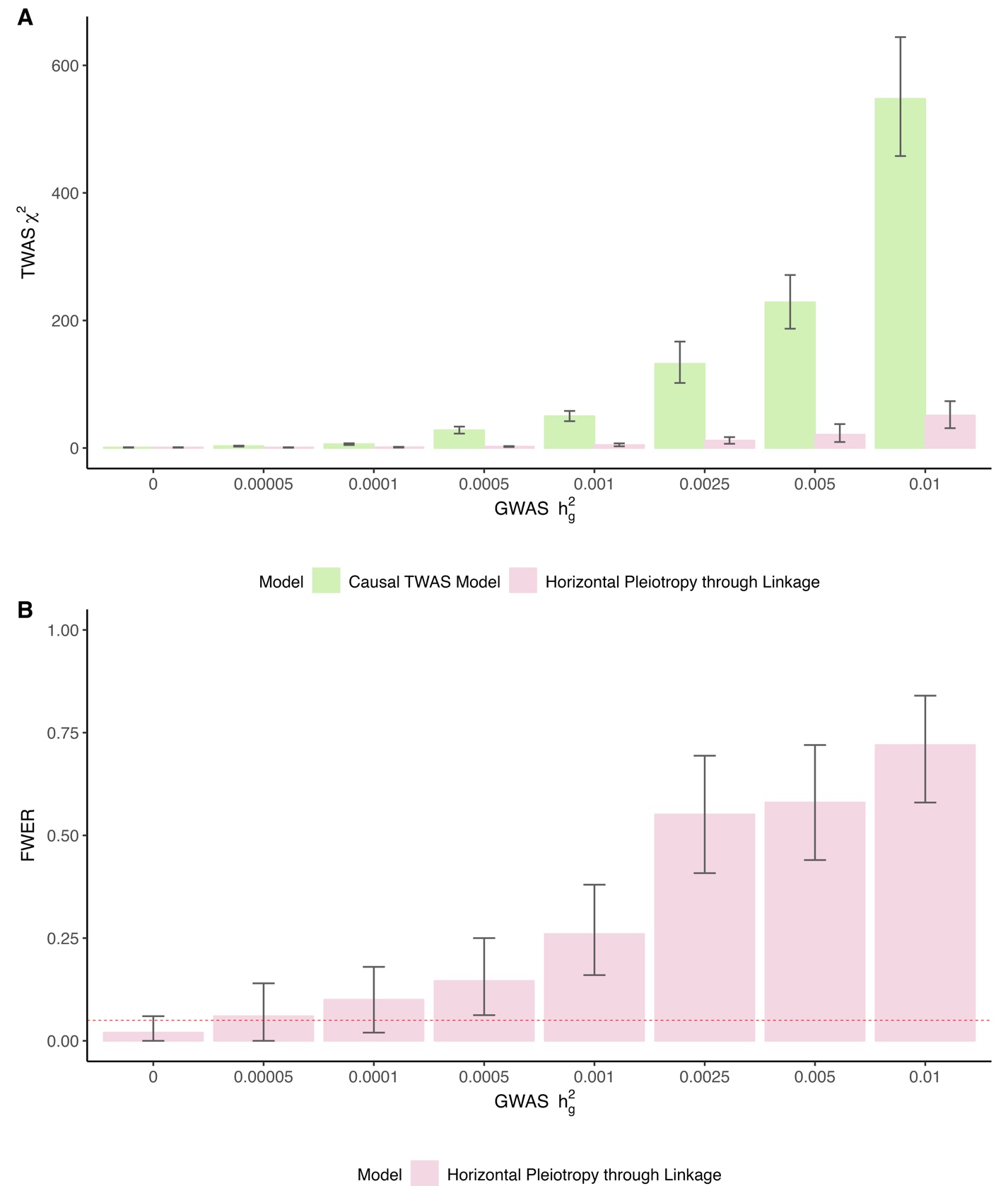
**

**Supplementary Figure 8.** **A.** **TWAS χ^2^ test-statistics generated from a model of horizontal pleiotropy through linkage as compared to TWAS model.** **B.** **FWER increased as GWAS** $\mathbf{h}_{\mathbf{g}}^{\mathbf{2}}$ **increase in horizontal pleiotropy through linkage model**. The bar plot represents TWAS χ^2^ test-statistics for canonical parameters with various GWAS $h_{g}^{2}$. 50 simulations were performed for each fixed set of simulation parameters. The TWAS χ^2^ and their error bars (95% confidence interval) are estimated using bootstraps with 1,000 repeats.

# Supplementary Tables

| **Label** | **Description** |
| --- | --- |
| gwas.sim | GWAS mode |
| real.time | Real time spent on the current simulation |
| cpu.time | CPU time spent on the current simulation |
| linear_model | Linear model used in the current simulation |
| h2ge | Variance explained in trait by components of GE |
| snp_model | SNP model used in the current simulation |
| nsnps | Number of SNPs |
| ngwas | GWAS sample size |
| nqtl | eQTL sample size |
| h2g | Narrow-sense heritability of GE |
| h2g.hat | Predicted narrow-sense heritability of GE |
| avg.ldsc | Average LD-score at the region |
| min.gwas.p | Minimum GWAS SNP p-value |
| mean.gwas.chi2 | Mean GWAS SNP chi-sq |
| median.gwas.chi2 | Median GWAS SNP chi-sq |
| twas.z | TWAS Z score |
| twas.p | TWAS p-value |
| alpha | TWAS alpha |

**Supplementary Table 1. Structure of TWAS simulator OUTPUT.summary.tsv** “Label” represents output file’s column names.

| **Column** | **Description** |
| --- | --- |
| chrom | Chromosome |
| snp | SNP identifier |
| pos | bp position |
| a0 | Non-effect allele |
| a1 | Effect allele |
| maf | Minor allele frequency |
| ld.score | LD score (ie. sum_i r_ij^2, where r_ij is LD between snps i, j) |
| ld.score.causal | LD score for causal variants |
| gwas.sim | GWAS mode |
| gwas.true | True causal effect for complex trait |
| gwas.beta | Beta coefficient in GWAS |
| gwas.se | Standard error in GWAS |
| eqtl.true | True causal effect for expression |
| eqtl.beta | Beta coefficient in eQTL |
| eqtl.se | Standard error in eQTL |
| eqtl.model | Linear model to predict gene expression from genotype |
| eqtl.model.beta | Coefficient estimated in selected linear model |

**Supplementary Table 2. Structure of TWAS simulator OUTPUT.scan.tsv** “Column” represents output file’s column names.

| Canonical parameters with various GWAS mode | | |
| --- | --- | --- |
| GWAS Mode | Statistics | P Value |
| Fast | 0.14 | 0.26 |
| Standard | 0.07 | 0.95 |
|  |  |  |
| Canonical parameters with various linear model | | |
| Linear Model | Statistics | P Value |
| Elastic Net | 0.14 | 0.26 |
| LASSO | 0.17 | 0.10 |
| GBLUP | 0.10 | 0.67 |
| True eQTL | 0.12 | 0.48 |
| External (SuSiE) | 0.16 | 0.16 |
|  |  |  |
| Canonical parameters with various SNP model | | |
| SNP Model | Statistics | P Value |
| 10% SNPs | 0.10 | 0.71 |
| 1% SNPs | 0.14 | 0.26 |
| 1 SNP | 0.12 | 0.45 |
|  |  |  |
| Canonical parameters with increasing eQTL sample size | | |
| eQTL Sample Size | Statistics | P Value |
| 100 | 0.10 | 0.75 |
| 250 | 0.14 | 0.26 |
| 500 | 0.16 | 0.14 |
|  |  |  |
| Canonical parameters with increasing GWAS sample size | | |
| GWAS Sample Size | Statistics | P Value |
| 50K | 0.12 | 0.51 |
| 100K | 0.14 | 0.30 |
| 200K | 0.14 | 0.26 |
| 500K | 0.12 | 0.46 |
|  | | |
| Canonical parameters with various reference panels | | |
| Reference Panel | Statistics | P Value |
| Full | 0.14 | 0.26 |
| Subset | 0.11 | 0.57 |
|  |  |  |
| Canonical parameters with various GWAS and eQTL signals | | |
| GWAS and eQTL signals | Statistics | P Value |
| Dependent GWAS and eQTL Signals | 0.11 | 0.57 |
| Independent GWAS and eQTL Signals | 0.11 | 0.59 |

**Supplementary Table 3. One-sample, two-sided Kolmogorov-Smirnov Test Statistics.**

# Supplementary Algorithms

**Supplementary Algorithm 1. External module to call susieR.** This Python script creates a subprocess to run custom R code (external.R; see Supplementary Algorithm 2), that imports simulated eQTL data to fit the susieR model and export fitted prediction weights. These fitted weights are imported and returned to the primary twas_sim module for use in TWAS testing.

"""

This file (`external_r.py`) is an example of how to define an external/custom function to

fit a predictive model of gene expression from genotype to be used by `twas_sim`. Here

we are illustrating how to call an external R script to call susieR on the simulated

data. Please see `external.R` for example R script details.

External modules -must- include a function named `fit` that takes as arguments:

Z: numpy matrix of genotype

y: numpy vector of gene expression/phenotype

h2g: the true h2g of gene expression

b_qtls: the true beta/effect-sizes for gene expression (i.e. eQTL)

args: the argparse object from twas_sim; useful for pulling `args.output`

as a prefix for temp data.

Similarly, it must return a tuple containing (coef, r2, logl):

coef: the numpy vector for estimated eQTL weights

r2: the predictive r2 (optional; None)

logl: the log likelihood of the model (optional; None)

"""

import subprocess

import numpy as np

def fit(Z, y, h2g, b_qtls=None, args=None):

# create output/input paths

geno_path = f"{args.output}.eqtl.genotype.txt.gz"

pheno_path = f"{args.output}.eqtl.gexpr.txt.gz"

coef_path = f"{args.output}.susie.coef.txt.gz"

# write genotype and phenotype to disk so that R can load it

np.savetxt(geno_path, Z, fmt="%.5f")

np.savetxt(pheno_path, y, fmt="%.5f")

# launch R script in a separate process

# R script reads in genotype, phenotype matrix and writes out SuSiE-inferred

# coefficients to `coef_path`

subprocess.run(

f"~/miniconda3/bin/Rscript external.R {geno_path} {pheno_path} {coef_path}",

shell=True,

check=True,

)

# load/read in SuSiE-inferred coefficients

coef = np.loadtxt(coef_path)

# r2 and logl are optional => hence `None`

return coef, None, None

# Supplementary Algorithm 2. R code to import simulated eQTL data and output susieR prediction weights.

# Example R script (external.R) to read in txt-based genotype data, along with

# gene expression data. Here, we fit SuSiE using the `susieR` package,

# pull coefficients at genotype (ignoring constant term)

# and write out results so that Python can read them in/load them.

library(susieR)

library(readr)

# paths to genotype, phenotype, and output

args <- commandArgs(trailingOnly=TRUE)

z_path <- args[1]

y_path <- args[2]

out_path <- args[3]

# load data as matrices

Z <- as.matrix(read.table(z_path, header = FALSE, dec = "."))

y <- as.matrix(read.table(y_path, header = FALSE, dec = "."))

# run SuSiE using at most 5 effects

res <- susie(Z, y, L=5)

# pull coefficients at genotypes (first coef is intercept)

g_coef <- data.frame(COEF=coef(res)[2:length(coef(res))])

# write out the result, ignoring column name

write_tsv(g_coef, out_path, col_names= FALSE)

# Supplementary References

1000 Genomes Project Consortium *et al.* (2015) A global reference for human genetic variation. *Nature*, **526**, 68–74.

Berisa,T. and Pickrell,J.K. (2016) Approximately independent linkage disequilibrium blocks in human populations. *Bioinformatics*, **32**, 283–285.

Gamazon,E.R. *et al.* (2015) A gene-based association method for mapping traits using reference transcriptome data. *Nat. Genet.*, **47**, 1091–1098.

Goeman,J.J. (2010) L1 penalized estimation in the Cox proportional hazards model. *Biom. J.*, **52**, 70–84.

Gusev,A. *et al.* (2016) Integrative approaches for large-scale transcriptome-wide association studies. *Nat. Genet.*, **48**, 245–252.

Hoerl,A.E. and Kennard,R.W. (1970) Ridge regression: Biased estimation for nonorthogonal problems. *Technometrics*, **12**, 55–67.

International HapMap 3 Consortium *et al.* (2010) Integrating common and rare genetic variation in diverse human populations. *Nature*, **467**, 52–58.

O’Leary,N.A. *et al.* (2016) Reference sequence (RefSeq) database at NCBI: current status, taxonomic expansion, and functional annotation. *Nucleic Acids Res.*, **44**, D733-45.

Pasaniuc,B. and Price,A.L. (2017) Dissecting the genetics of complex traits using summary association statistics. *Nat. Rev. Genet.*, **18**, 117–127.

Patterson,H.D. and Thompson,R. (1971) Recovery of Inter-Block Information when Block Sizes are Unequal. *Biometrika*, **58**, 545.

Searle,S.R. *et al.* (1992) Variance Components Searle,S.R. *et al.* (eds) John Wiley & Sons, Nashville, TN.

Shi,H. *et al.* (2016) Contrasting the genetic architecture of 30 complex traits from summary association data. *Am. J. Hum. Genet.*, **99**, 139–153.

Sudmant,P.H. *et al.* (2015) An integrated map of structural variation in 2,504 human genomes. *Nature*, **526**, 75–81.

Tibshirani,R. (1996) Regression shrinkage and selection via the lasso. *J. R. Stat. Soc.*, **58**, 267–288.

Wang,G. *et al.* (2020) A simple new approach to variable selection in regression, with application to genetic fine mapping. *J. R. Stat. Soc. Series B Stat. Methodol.*, **82**, 1273–1300.

Yang,J. *et al.* (2012) Conditional and joint multiple-SNP analysis of GWAS summary statistics identifies additional variants influencing complex traits. *Nat. Genet.*, **44**, 369–75, S1-3.

Zhu,X. and Stephens,M. (2017) Bayesian large-scale multiple regression with Summary Statistics from genome-wide association studies. *Ann. Appl. Stat.*, **11**, 1561–1592.

Zou,H. and Hastie,T. (2005) Regularization and variable selection via the elastic net. *J. R. Stat. Soc. Series B Stat. Methodol.*, **67**, 301–320.
